# Metabolic heterogeneity caused by HLA-DRB1*04:05 and protective effect of inosine on autoimmune hepatitis

**DOI:** 10.3389/fimmu.2022.982186

**Published:** 2022-08-05

**Authors:** Fan Yang, Leyu Zhou, Yi Shen, Shenglan Zhao, Yanyi Zheng, Ruoting Men, Xiaoli Fan, Li Yang

**Affiliations:** Sichuan University-University of Oxford Huaxi Joint Centre for Gastrointestinal Cancer, Department of Gastroenterology and Hepatology, West China Hospital, Sichuan University, Chengdu, China

**Keywords:** autoimmune hepatitis, HLA-DRB1*04:05, metabolomics, inosine, CD4^+^ T cell

## Abstract

Autoimmune hepatitis (AIH) is an autoimmune disease caused by disruption of liver immune homeostasis. Genetic studies have revealed the predisposition of AIH with the human leukocyte antigen (HLA) region. Recently, metabolomics integrated with genomics has identified many genetic loci of biomedical interest. However, there is no related report in AIH. In the present study, we found that HLA-DRB1*04:05 was linked to the clinical features and prognosis of AIH in Chinese patients. Furthermore, our patients were divided into DRB1*04:05 positive and DRB1*04:05 negative groups and the metabolic profiling was done by HPLC/MS. We chose inosine, one of the highly altered metabolites, to explore the effect on an acute severe hepatitis murine model. The results showed that inosine treatment attenuated hepatocyte apoptosis, enhanced antioxidant ability and inhibited the activation and glycolysis of CD4^+^ T cell. We propose that inosine participates in the regulation of AIH through its protective effect on hepatocytes and inhibition of overactivated immune cells, which might provide a potential novel approach in treating acute form of AIH.

## Introduction

Autoimmune hepatitis (AIH) is a rare chronic progressive liver disease characterized by antibodies, hypergammaglobulinemia, and interface hepatitis. Although the etiology and pathogenesis remain unclear, the genetic and environmental factors play an important role in the development of the disease. Genetic studies have found that genetic predisposition to AIH is associated with the human leukocyte antigen (HLA) region (1). Increasing research has identified HLA-DR3 or -DR4 as the most convincing disease susceptibility locus (2). HLA-DRB1*03:01 and DRB1*04:01 are associated with AIH in European populations (3). HLA-DRB1*04:05 is considered to increase susceptibility to AIH in Japanese and Korean populations (4–6). The results showed that DRB1*04:05 was significantly associated with elevated serum IgG and anti-smooth muscle antibody positivity, which established the role of HLA in the progression of AIH. However, the precise mechanisms have not been sufficiently revealed.

Metabolomics has developed rapidly as a new omics technique after genomics, transcriptomics, and proteomics. Metabolites are intermediates and final products of cellular activities, and their levels can be considered the result of the interaction between the genome, transcriptome, and proteome (7). To date, many disease-related metabolomics have been performed to define the different metabolites between each group and facilitate the characterization of different pathological conditions (8–11). Li et al. identified several cirrhosis-associated metabolites in AIH and revealed the potential of drug regulation metabolism in the treatment of AIH (10). Recently, the integration of genetics and metabolomics has identified many genetic loci of biomedical interest (12). Kirchberg et al. conducted a prospective study to investigate the association between the metabolic profile and HLA-risk in celiac disease patients (11). However, there is no report on the difference in metabolites of AIH patients related to the HLA allele.

**Figure 1:**
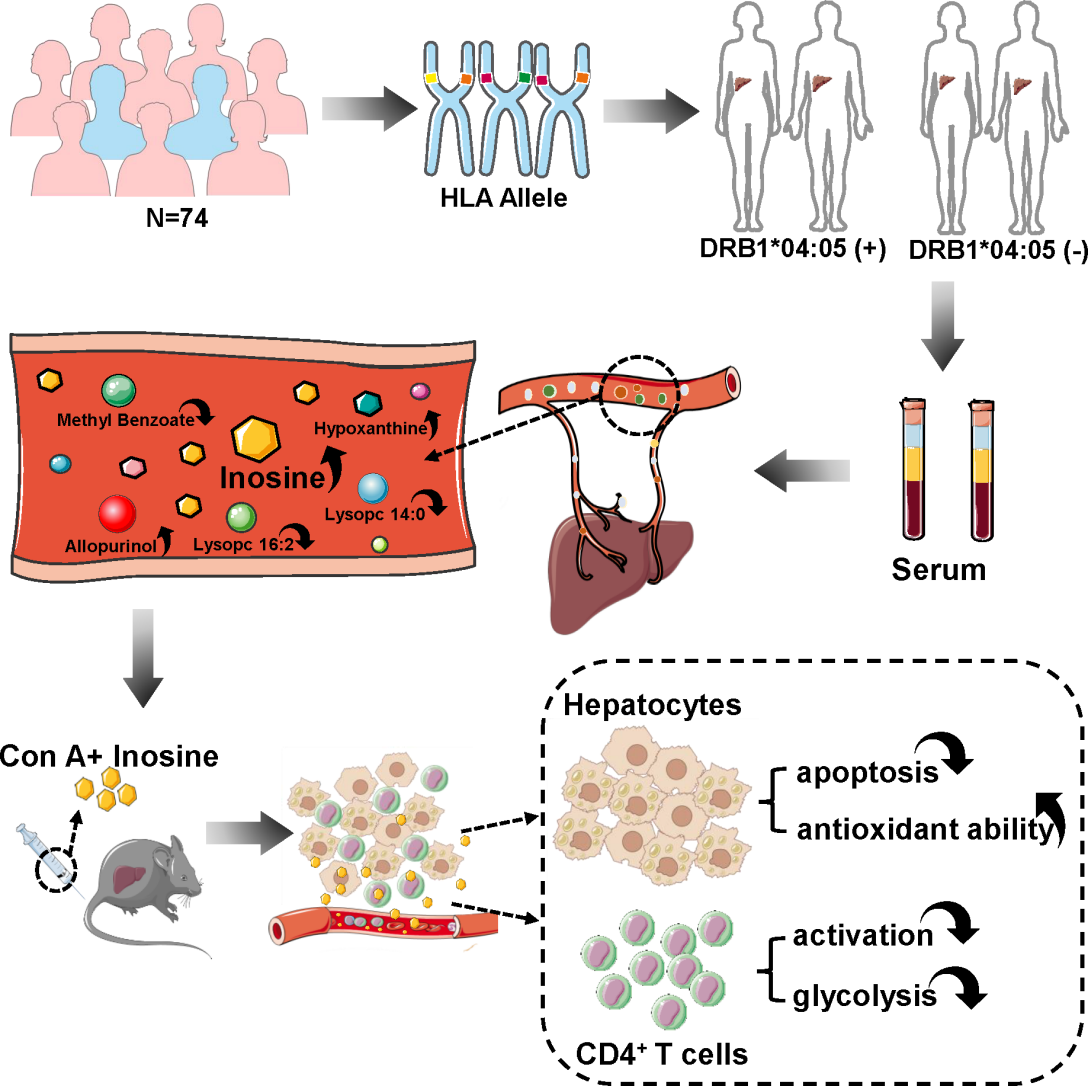
Overview of this study. 74 patients were recruited and their blood was collected for HLA genotyping. These patients were divided into DRB1*04:05 positive and negative groups to detect serum metabolic profiles. Inosine is a highly alerted metabolite. Con A-induced hepatitis murine model was used to explore the effect of inosine. After injection, inosine treatment was found to attenuate hepatocyte apoptosis, enhance antioxidant ability, and inhibit the activation and glycolysis of CD4^+^ T cells.

In this study, we performed HLA-DRB1 genotyping on Chinese AIH patients and divided the patients into DRB1*04:05 positive and negative groups. HPLC/MS was used to detect the difference in metabolic profiles between the two groups. Inosine, reported as a metabolite with immunomodulatory effects (13, 14), was found to be highly altered between the DRB1*04:05 positive and negative groups. There are few reports on inosine in AIH. To explore the effect of inosine, we used a concanavalin A (Con A)-induced murine model. Inosine treatment ameliorated liver damage by attenuating hepatocyte apoptosis and increasing antioxidant ability. Furthermore, inosine inhibited the activation and glycolysis of CD4^+^ T cells to impede the inflammatory response (**Figure 1**). The current study revealed HLA DRB1*04:05 related metabolic heterogeneity and provided the potential function of inosine in acute form of AIH.

## Materials and methods

### Patients and healthy volunteers

In the present study, a total of 74 peripheral blood samples were collected from patients with AIH between January 2019 and November 2021 at the West China Hospital of Sichuan University. The diagnostic criteria adhered to the International Autoimmune Hepatitis Group (1999) guidelines (15, 16). Among all patients, 72 patients had AIH-1 and 2 patients had AIH-2. Peripheral whole blood was available for HLA genotyping (Weihe Biotechnology Inc. Nanjing, China). The clinical characteristics of these patients were listed in **Supplementary Table S1**. In addition, 48 peripheral blood samples were randomly selected to detect metabolic profiles and their clinical characteristics were listed in **Supplementary Table S2**. The whole study was approved by the Independent Ethics Committee of West China Hospital and conducted in accordance with the relevant principles.

### HPLC−MS/MS analysis

Serum from patients was prepared by centrifugation of whole blood at 2000 g for 10 min., placed in the Eppendorf tubes and resuspended in prechilled 80% methanol by well vortexing. Then the samples were incubated on ice for 5 min and centrifuged at 15000 g and 4°C for 20 min. Subsequently, the samples were transferred to a fresh Eppendorf tube, centrifuged at 15000 g for 20 min, and injected into the LC-MS/MS system for analysis. LC−MS/MS analyses were performed using an ExionLC™ AD system (SCIEX) coupled with a QTRAP^®^ 6500+ mass spectrometer (SCIEX) in Novogene Co., Ltd. (Beijing, China). MRM (multiple reaction monitoring) was used to detect the experimental samples. These metabolites were annotated using the KEGG database (http://www.genome.jp/kegg/), HMDB database (http://www.hmdb.ca/) and Lipidmaps database (http://www.lipidmaps.org/). Principal component analysis (PCA) and partial least squares discriminant analysis (PLS‐DA) were performed at metaX. The metabolites with VIP > 1.0 and P value < 0.05 and fold change > 1.2 or < 0.833 were regarded as differential metabolites. The functions of these metabolites and metabolic pathways were studied using the KEGG database.

### Animals

Female C57BL/6J mice (aged 8-10 weeks; weighing 20-22 g) were obtained from the Experimental Animal Center of Sichuan University (Chengdu, China). All animal experiments were approved by the Animal Ethics Committee of West China Hospital, Sichuan University. The ConA-induced murine model was established according to the previous study (17). After 7 days of adaptive feeding, all mice were randomly assigned to three groups as follows: (1) NC group: mice were given an intravenous injection of sterile saline as a control; (2) Con A group: mice were administrated Con A (Sigma−Aldrich, St. Louis, MO, United States) intravenously at a dose of 10 mg/kg body weight; (3) Con A + Inoine (Ino) group: Mice were injected with 300mg/kg body weight inosine (MedChemExpress, Monmouth Junction, NJ, USA) and administered with an equal volume of Con A. Inosine was dissolved in sterile saline. All mice were sacrificed at 24 h after injection for sample collection.

### Cell culture

Splenic mononuclear cells were isolated from mice by mouse lymphocyte separation medium (Dakewei, Shenzhen, China) as previously described (17). Single-cell suspensions were separated by mechanical disruption of mouse spleens through 70-μm cell strainers and were harvested by gradient centrifugation at 800 g for 30 min, and washed in PBS twice for the experiment. Purified CD4^+^ T cells were negatively selected using the MojosortTM Mouse CD4^+^ T Cell Isolation kit (Biolegend, San Diego, CA, USA). The purity of CD4^+^ T cell was > 95% (**Supplementary Figure S1A**), and the cells were cultured in RMPI 1640 medium with recombinant murine IL-2 (20 IU/ml, Novoprotein, Shanghai, China). The cells were stimulated with plate-bound anti-CD3 (2.5 µg/ml, LEAF™ Purified anti-mouse CD3ϵ, Biolegend, San Diego, USA) and soluble anti-CD28 (2.5 µg/ml, LEAF™ Purified anti-mouse CD28, Biolegend, San Diego, USA), and the cells were incubated for 24 h for further experiments.

### Flow cytometric analysis

The liver mononuclear cells were collected and suspended in PBS as described previously (18). In general, the liver cell suspension was centrifuged at 30 g for 5 min. Supernatants were collected, washed in PBS, and resuspended in 40% Percoll (Solarbio, Beijing, China). Furthermore, the cell suspension was gently overlaid onto 70% Percoll and centrifuged for 20 min at 800 g. Liver mononuclear cells were collected from the interphase, washed twice in PBS, and resuspended in PBS for FACS analysis. The expression of cell surface molecules was detected by staining with antibodies against CD3 (APC-Cy7, Biolegend, San Diego, USA), CD4 (FITC, Biolegend, San Diego, USA), CD8 (Percp-Cy5.5, Biolegend, San Diego, USA), CD25 (PE, Biolegend, San Diego, USA), CD69 (APC, Biolegend, San Diego, USA), and DAPI (Biolegend, San Diego, USA), diluting each antibody according to the manufacture’s instruction.

### Liver function assay and cytokine assay

Retroorbital blood samples were collected from mice in each group and centrifuged at 1000 g for 10 min. Alanine aminotransferase (ALT) and aspartate transaminase (AST) levels were detected by an automatic dry biochemical analyzer (Hitachi, Tokyo, Japan). The levels of IFN-γ and TNF-α were measured using mouse ELISA detection kits according to the manufacturer’s recommendations (Dakewei, Shenzhen, China). The final density values were measured at 450 and 570 nm by a microplate reader (BioTek, Winooski, VT, United States). In addition, the supernatant of the cell culture was collected by centrifugation at 300 g for 10 min. The levels of cytokines were analyzed by ELISA kits according to the manufacturer’s instructions (MultiSciences, Hangzhou, China).

### Histopathology assay and tunnel staining

Liver tissues were fixed in 4% formalin and embedded in paraffin. The samples were sliced into 3-4 μm sections, followed by dewaxing and rehydration. Furthermore, the sections were stained with hematoxylin and eosin (H&E) to observe the level of tissue damage by light microscopy (Servicebio, Wuhan, China). Six optional fields per section were selected to calculate the histological score. The injury score (Suzuki’s criteria) is recognized as the degree of liver injury with three indicators, including sinusoidal congestion, hepatocyte necrosis and ballooning degeneration (19). TUNEL staining was carried out with a TUNEL BrightRed Apoptosis Detection Kit (Biossci, Wuhan, China) and observed under a fluorescence microscope. The average number of TUNEL^+^ cells in six fields of each section was counted and used to calculate the ratio of apoptotic cells. All the slides were evaluated by at least three professional researchers in a double-blind assessment.

### Measurement of oxidative and antioxidant biomarkers

The serum concentration of malondialdehyde (MDA) was measured by an MDA assay kit (Beyotime Biotech, Shanghai, China) according to the manufacturer’s instruction. The serum level of Fe^2+^ was determined by a spectrophotometric method and quantified at a wavelength of 586 nm. The ratio of reduced glutathione (GSH) and oxidized glutathione (GSSG) was detected by a commercial GSSG/GSH quantification kit (DOJINDO, Tokyo, Japan). The luminescence of all above was detected by the luminometer (PerkinElmer, MA, United States).

### Cell proliferation

Cell proliferation was measured using the CCK-8 assay. CD4^+^ T cells were seeded in 96-well plates at 500,000 cells/well and stimulated with CD3/CD28. After 24 stimulations, 20 ul CCK-8 reagent (Beyotime Biotech, Shanghai, China) was added to each well and incubated for 4 h at 37°C away from light. The optical density (OD) values were determined using a microplate reader at 450 nm. Each assay was performed in triplicate.

### Lactic acid assay and ATP concentration

The lactic acid level from each sample was measured using a commercial kit (Jiancheng, Nanjing, China) according to the manufacture’s instruction. The ATP concentration was detected with an ATP assay kit (Beyotime Biotech, Shanghai, China). The luminescence of each sample was detected by the luminometer (PerkinElmer, MA, United States).

### Glucose uptake assay

CD4^+^ T cells were stimulated for 24 hours in the presence or absence of inosine. Moreover, the cells were washed twice in PBS and incubated at 37 °C for 20 minutes in PBS containing 2-NBDG (0.1-0.3 mM) before analysis using flow cytometry.

### Quantitative Real-Time PCR

Total RNA was extracted from liver tissues and cells in each group using TRIzol reagent (Tiangen, Beijing, China). An Agilent 2100 Bioanalyzer (Agilent Technologies, Santa Clara, CA, USA) and Qubit Fluorometer (Invitrogen) were used to assess RNA quality. cDNA was synthesized using a PrimeScrip RT reagent kit (Takara, Shiga, Japan), and all primers were obtained from Tsingke (Beijing, China). RT−qPCR was performed using SYBR Green Supermix on a CFX96 RT−qPCR detection system (BioRad, Hercules, CA, United States). The expression levels of ncRNAs were normalized to actin expression level.

### Western blot analysis

Protein extraction from each sample was performed using radioimmunoprecipitation assay (RIPA) buffer containing protease and phosphatase inhibitors. The protein concentration was determined by a BCA kit (Beyotime,Biotech, Shanghai, China). Equivalent amounts of total protein were separated on an SDS−PAGE gel and transferred to polyvinylidene difluoride (PVDF) membranes. The membranes were blocked in 5% nonfat milk at RT for 1h. Then, the PVDF membranes were incubated with NLRP3 (Adipogen, Inc), Caspase 3 (Abcam, Inc.), GPX4 (Abcam, Inc), PFKFB3 (Abcam, Inc.), HK2 (Abcam, Inc), GLUT1 (Huabio,Inc) and β-actin (Cell Signaling Technology, Inc) overnight at 4°C. The blots were washed and incubated with an HRP-conjugated secondary antibody at 37°C for 1 hour. Finally, the membranes were washed twice and detected by a chemiluminescence kit. Protein quantification was carried out using ImageJ software (NIH) and relative to β-actin expression.

### Statistical analysis

All data are presented as the mean ± SD or median. and were statistically analyzed with GraphPad Prim 9 (GraphPad Software Inc., San Diego, CA, United States) and SPSS 28.0 software (SPSS Inc., Chicago, IL, United States). Student’s t-test, one-way ANOVA, the Mann–Whitney *U* test, and the chi-square test were performed to compare the differences between two groups or intergroup differences. Statistical significance was labeled according to the p value as *p value < 0.05, **p value < 0.01, and ***p value < 0.001.

## Results

### HLA-DRB1*04:05 allele associated with the clinical features of AIH patients

Genetic susceptibility to AIH is strongly associated with HLA genes and HLA-DRB1*04:05 was considered to increase the susceptibility of AIH in Asian populations (2, 20). To explore the relationship between the HLA-DRB1*04:05 allele and disease course in Chinese AIH patients, we performed HLA-DRB1 genotyping on 74 adult AIH patients. The allele frequency of HLA-DRB1*04:05 was 14.9%, and 22 patients were HLA-DRB1*04:05 positive (**Table 1**). Furthermore, we divided the patients into HLA-DRB1*04:05 positive group and HLA-DRB1*04:05 negative group. The clinical characteristics between the two groups are compared in **Table 2**. The age and proportion of females were similar between the two groups. For laboratory examination, no significant difference was found in ALT, AST, ALP, GGT, ALB, or IgM. Higher serum levels of IgG and GLB were seen in the HLA-DRB1*04:05 positive group than in the negative group. The positive rate of ANA was higher in DRB1*04:05 positive patients than in DRB1*04:05 negative patients, but the difference did not reach statistical significance (P=0.061). The positive rate of SLA was higher in the DRB1*04:05 positive group than in the DRB1*04:05 negative group (P=0.045), which is associated with disease severity (21). For histological examination (inflammation grade (G) and fibrosis stage (S)), there was no significant difference between the two groups. However, the IAIHG score was higher in DRB1*04:05 positive patients than in DRB1*04:05 negative patients(P=0.046). Although the rate of patients achieving complete biochemical remission within 6 months was similar between the two groups, the time to remission was longer in the DRB1*04:05 positive group than in the DRB1*04:05 negative group (6.3 (2.0, 23.6) vs. 4.0 (0.2, 16.7), P=0.046). This may suggest that HLA-DRB1*04:05 was associated with response to treatment. Moreover, the frequency of other extrahepatic autoimmune diseases, such as Sjogren’s syndrome (SS) and rheumatoid arthritis (RA), was higher in DRB1*04:05 positive patients than in DRB1*04:05 negative patients (36.4% vs. 7.7%, P=0.007). Some studies revealed an association between the progression of joint destruction and HLA-DRB1*04:05 in RA (22). Recently, increasing research has focused on the effect of genetic variations on the metabolic pathways (11). However, there have been few reports on AIH. Therefore, we divided the patients into groups according to the DRB1*04:05 allele to determine the serum metabolite profile.

**Table 1:** Frequencies of HLA-DRB1 alleles in autoimmune hepatitis.

DRB1 alleles	AIH patients n = 74	DRB1 alleles	AIH patients n = 74
01:01	2% (3)	12:01	2.7% (4)
03:01	16.9% (24)	12:02	7.4% (10)
04:01	2% (3)	13:02	1.4% (2)
04:03	0.7% (1)	13:12	2.7% (4)
04:05	14.9% (22)	14:01	4.1% (6)
04:06	0.7% (1)	14:04	1.4% (2)
04:11	0.7% (1)	14:05	2.0% (3)
07:01	2.7% (4)	14:54	0.7% (1)
08:03	6.8% (10)	15:01	8.1% (11)
09:01	12.2% (18)	15:02	2.0% (3)
10:01	2% (3)	16:02	4.1% (6)
11:01	2% (3)		

**Table 2:** Clinical characteristics of HLA-DRB1*04:05 positive and HLA-DRB1*04:05 negative AIH patients at diagnosis.

Features	DRB1*04:05 (+) n = 22	DRB1*04:05 (-) n = 52	P value
Age (years)	56 (25, 74)	52 (26, 75)	0.574
Gender (M:F)	5:17	7:45	0.520
ALT (IU/L)	210 (23, 1904)	201 (22, 1543)	0.808
AST (IU/L)	261 (36, 1585)	235 (26, 1751)	0.896
ALP (IU/L)	148 (80, 256)	150 (59, 598)	0.428
GGT (IU/L)	138 (42, 764)	121 (17, 1003)	0.948
ALB (g/L)	35.8 ± 5.3	37.7 ± 5.8	0.183
GLB (g/L)	44.6 ± 11.4	38.5 ± 7.5	0.029
IgG (g/L)	29.3 ± 11.0	23.3 ± 7.3	0.025
IgM (mg/L)	1680 (251, 5670)	1470 (411, 8400)	0.696
ANA			0.061
<1:100	1	14	
≥1:100	21	38	
SLA			0.045
−	17	47	
+	5	2	
G			0.136
0~2	6	23	
3~4	16	27	
S			0.261
0~2	15	27	
3~4	7	23	
Pretreatment IAIHG score	18 (10, 24)	14 (10, 21)	0.040
Complete biochemical remission within 6 months n (%)	9 (40.9%)	27 (51.9%)	0.386
Time to achieve biochemical remission (months)	6.3 (2.0, 23.6)	4.0 (0.2, 16.7)	0.046
Extrahepatic autoimmune disorders	8 (36.4%)	4 (7.7%)	0.007

HLA, human leukocyte antigen; AIH, autoimmune hepatitis; ALT, alanine aminotransferase; AST, aspartate transaminase; ALP, alkaline phosphatase; GGT, gamma-glutamyl transferase; ALB, albumin; GLB, globulin; IgG, immunoglobulin G; IgM, immunoglobulin M; ANA, antinuclear antibody; SLA, soluble liver antigen; G, inflammation grade; S, fibrosis stage; IAIHG, international autoimmune hepatitis group.

### Patients with positive and negative DRB1*04:05 expression showed differences in metabolic profiles

**Figure 2:**
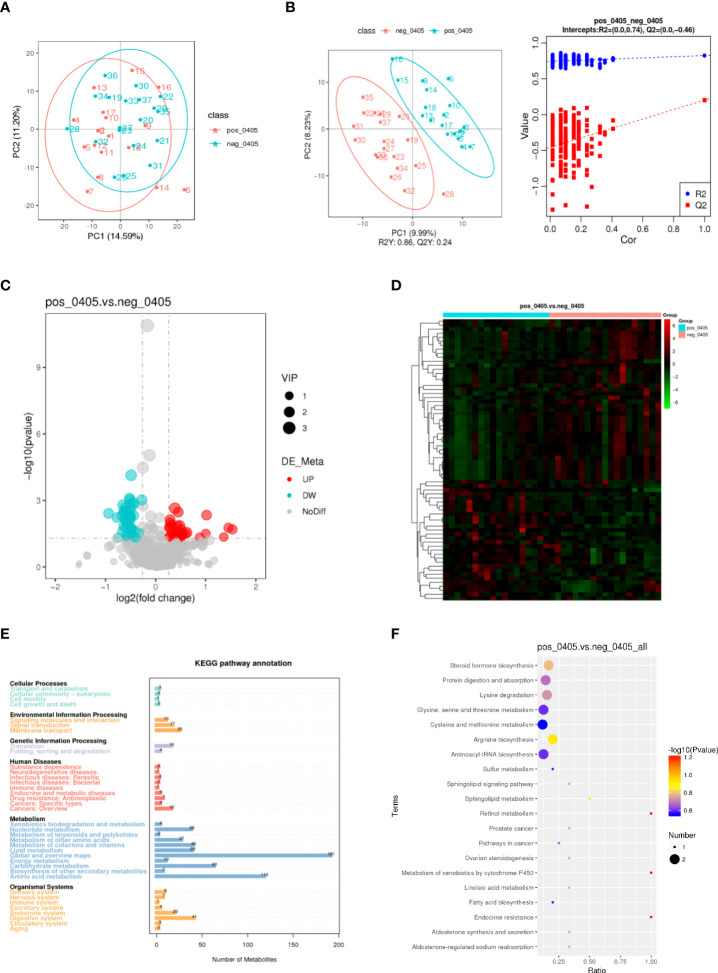
Metabolic profiles of DRB1*04:05-positive and -negative patients. **(A)** PCA score scatter plot of metabolites obtained from LC/MS. **(B)** PLS-DA score plots and validation plots showing separation of metabolites of the two groups. **(C, D)** Volcano plot and heatmap showing differences in metabolites between the two groups. **(E)** KEGG pathway annotations. **(F)** KEGG enrichment scatterplot showing the top 20 metabolic pathways.

To investigate the influence of the DRB1*04:05 allele on metabolic changes, we collected serum from DRB1*04:05 positive and DRB1*04:05 negative patients and performed metabolic profiling by LC/MS. As shown in **Figure 2A**, principal component analysis (PCA) revealed a trend of discrimination between DRB1*04:05 positive and negative patients. The partial least squares discriminant analysis (PLS-DA) score plot indicated the distinct separation of the two groups. Moreover, no obvious overfitting was observed in the permutation test (**Figure 2B**). Metabolites with VIP > 1.0 and P value < 0.05 and fold change > 1.2 or < 0.833 were regarded as differential metabolites. We first identified 70 metabolites with significant differences between the DRB1*04:05 positive and DRB1*04:05 negative group (**Figure 2C**). A heatmap was constructed based on the differential metabolites between the two groups and revealed clearing clustering (**Figure 2D**). Additionally, the functions of all metabolites and metabolic pathways were studied using the KEGG database. The main pathways involved were confirmed to be metabolism, including lipid metabolism, amino acid metabolism, carbohydrate metabolism and nucleotide metabolism (**Figure 2E**). The KEGG enrichment scatterplot showed the top 20 pathways based on the differentially altered metabolites (**Figure 2F**). According to **Table 3**, the top 10 most highly altered metabolites were mainly in nucleotides and their derivatives, including inosine, hypoxanthine-9-beta-D-Arabinofuranoside, hypoxanthine and allopurinol. Increasing evidence demonstrates that inosine possesses a wide range of anti-inflammatory and immunomodulatory properties (14, 23). Thus, we further explored the effect of inosine on the Con A- induced murine model.

**Table 3:** The top 10 metabolites with the most obvious changes.

Name	Class	log2FC	P value	VIP
Glucarate O-Phosphoric Acid	Carbohydrates And Its Derivatives	1.532529963	0.020129482	1.626484086
Inosine	Nucleotide And Its Derivates	1.451125765	0.016981841	1.570893577
Hypoxanthine-9-beta-D-Arabinofuranoside	Nucleotide And Its Derivates	1.358837416	0.044542092	1.342088144
Hypoxanthine	Nucleotide And Its Derivates	1.01556013	0.004595258	1.877520914
Allopurinol	Nucleotide And Its Derivates	1.000665369	0.031138467	1.434797824
Lysopc 16:2	Phospholipid	-0.940409983	0.003823747	1.968538981
Dulcitol	Carbohydrates And Its Derivatives	0.880385746	0.046832344	1.546999895
Lysopc 20:0	Phospholipid	-0.771378043	0.005859601	1.862457189
Lysopc 14:0	Phospholipid	-0.742518756	0.0464681	1.700699346
Methyl Benzoate	Benzoic Acid And Its Derivatives	-0.669830793	0.004902561	1.980387329

### Inosine treatment attenuated hepatocyte apoptosis and increased antioxidant ability in Con A-induced murine model

**Figure 3:**
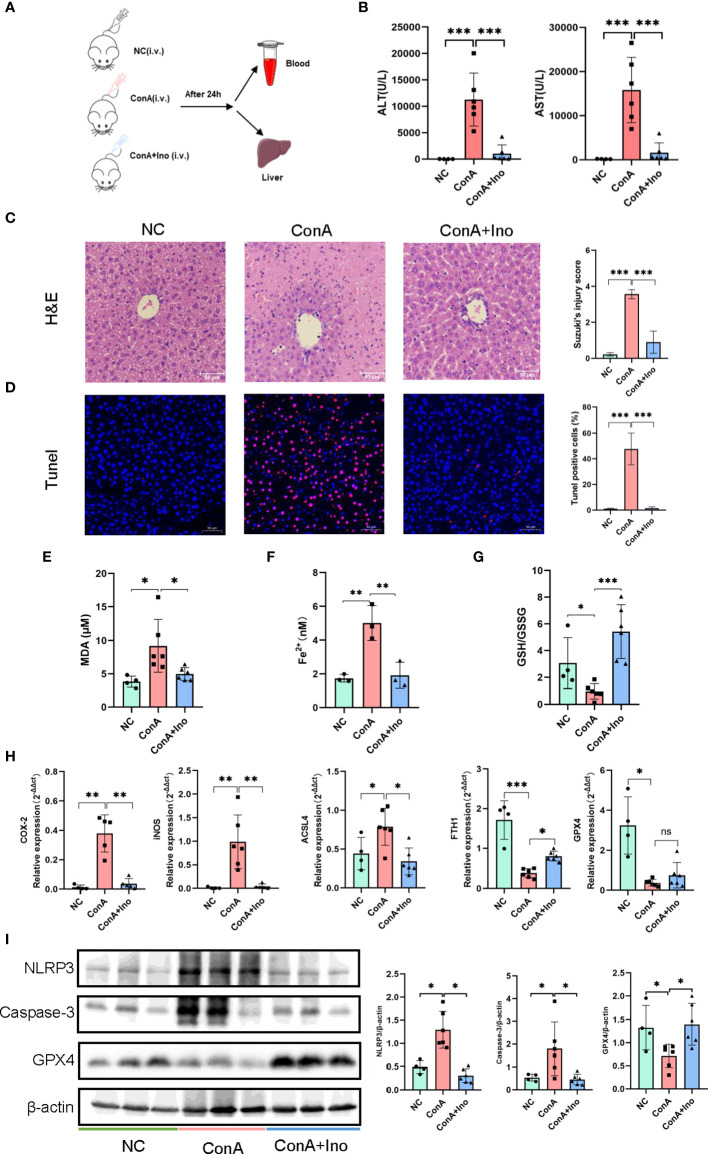
The effect of inosine on Con A-induced murine model. **(A)** Animal experimental design (N = 4-6). **(B)** ALT and AST levels. **(C)**. H&E staining. **(D)** TUNEL assays. **(E)** Serum MDA levels. **(F)** Serum Fe^2+^ levels. **(G)** The ratio of GSH/GSSG. **(H)** The gene expression of COX2, iNOS, ACSL4, FTH1 and GPX4, normalized to β-actin. **(I)** the protein expression of NLRP3, Caspase-3 and GPX4, relative to β-actin expression. The figure only shows some of the samples, but protein quantification was calculated for all samples in each group. *p value < 0.05, **p value < 0.01, and ***p value < 0.001.

Con A-induced hepatitis is characterized by lymphocyte-induced liver damage (24). To investigate the effect of inosine treatment on the Con A-induced murine model, all mice were sacrificed at 24 h after Con A injection (**Figure 3A**). The serum ALT and AST levels in the mice were measured. Compared to the NC group, the serum ALT and AST levels were dramatically elevated in the Con A group, but inosine treatment significantly decreased the AST and ALT levels (**Figure 3B**). In addition, H&E staining and TUNEL assays were performed. As shown in **Figure 3C**, there were more massive necrotic areas, severe sinusoidal congestion and increased inflammation were observed in the Con A group than in the NC group. However, inosine treatment clearly ameliorated these pathological symptoms. Moreover, the proportion of TUNEL^+^ cells in the Con A group was significantly larger than that in the NC group. However, treatment with inosine significantly reduced the proportion of apoptotic cells (**Figure 3D**). Apoptosis and inflammation-related proteins were also analyzed in the three groups. Compared to that in the NC group, the expression of Caspase-3 and the nod-like receptor pyrin domain 3 (NLRP3) inflammasome were increased in the Con A group but decreased in the Con A + Inosine group (**Figure 3I**). These results showed that inosine treatment clearly attenuated hepatocyte apoptosis.

To further assess the antioxidant effect of inosine treatment on Con A-induced hepatitis, the serum levels of MDA, Fe^2+^ and the GSH/GSSG ratio were measured. In contrast to the NC group, the MDA, and Fe^2+^ contents were increased in the Con A group. Inosine treatment significantly decreased the levels of MDA and Fe^2+^ (**Figures 3E, F**). Furthermore, compared to the NC group, the GSH/GSSG ratio was decreased in the Con A group and was clearly reversed by inosine treatment (**Figure 3G**). Moreover, the expression levels of mRNAs related to lipid peroxidation were analyzed. As shown in **Figure 3H**, the hepatic mRNAs expression of cyclooxygenase-2 (COX-2), inducible nitric oxide synthase (iNOS) and acyl-CoA synthetase long-chain family member 4 (ACSL4) were upregulated in the Con A group compared to the NC group, while treatment with inosine dramatically attenuated these adverse changes. Furthermore, inosine treatment increased the expression level of ferritin heavy chain 1 (FTH1). Although the mRNA expression level of glutathione peroxidase 4 (GPX4) in the inosine group had an increasing trend but without significance, treatment with inosine recovered GPX4 protein expression (**Figure 3I**). All these data suggested that inosine alleviated lipid peroxidation and increased antioxidant ability in Con A-treated mice.

### Treatment with inosine inhibited immune activation and prevented the inflammatory response in Con A-induced hepatitis

**Figure 4:**
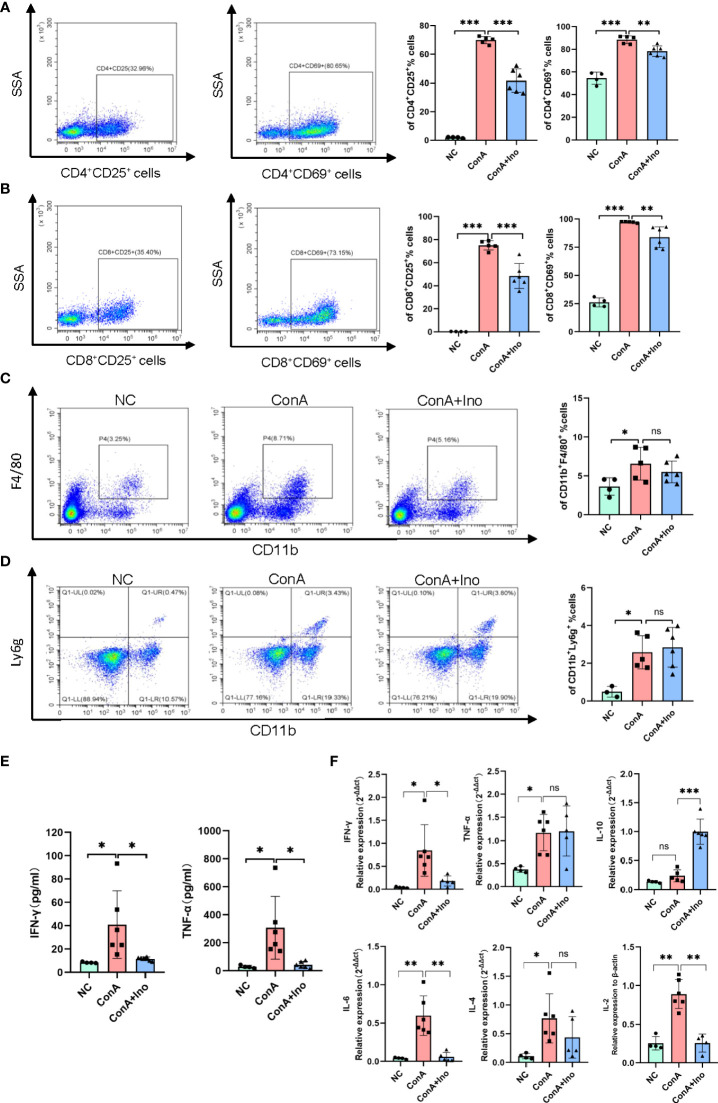
Effect of inosine on the inflammatory response. **(A-D)**. Flow cytometric analysis of the frequency of CD4^+^CD25^+^ T cells, CD8^+^CD25^+^ T cells, CD4^+^CD69^+^ T cells, CD8^+^CD69^+^ T cells, CD11b^+^F4/80^+^ cells and CD11b^+^Ly6g^+^ cells. **(E)** Serum levels of IFN-γ and TNF-α. **(F)** The gene expression of IFN-γ, TNF-α, IL-10, IL-6, IL-4, and IL-2, normalized to β-actin. *p value < 0.05, **p value < 0.01, and ***p value < 0.001.

Con A-induced hepatitis is a T-cell mediated murine model, accompanied by the activation of immune cells and the release of many cytokines. To investigate the effect of inosine treatment on immune cells, we detected the frequency of activated T cells, as well as macrophages and neutrophils in the livers of Con A-induced mice by flow cytometry. The results showed that the frequencies of hepatic CD4^+^CD25^+^, CD4^+^CD69^+^, CD8^+^CD25^+^ and CD8^+^CD69^+^ T cells were significantly increased after Con A injection when compare to normal control, along with the frequencies of CD11b^+^F4/80^+^ cells and CD11b^+^Ly6g^+^ cells (**Figures 4A–D**). The gating strategy was shown in **Supplementary Figure S1A**. Treatment with inosine greatly decreased the frequencies of CD4^+^CD25^+^ T cells, CD8^+^CD25^+^ T cells, CD4^+^CD69^+^ T cells and CD8^+^CD69^+^ T cells (**Figures 4A, B**). However, there were no significant differences observed for the frequencies of CD11b^+^F4/80^+^ cells and CD11b^+^Ly6g^+^ cells between the Con A group and the Con A + inosine group (**Figures 4C, D**). These results suggested that inosine might ameliorate Con A-induced hepatitis by suppressing the activation of CD4^+^ T cells and CD8^+^ T cells. Furthermore, the serum IFN-γ and TNF-α levels were measured to assess the inflammatory response in Con A-induced mouse model. Compared to the NC group, there was a dramatic elevation in the serum IFN-γ and TNF-α levels in the Con A group. However, treatment with inosine clearly suppressed this change in IFN-γ and TNF-α levels (**Figure 4E**). The expression levels of mRNAs related to inflammation were determined. As shown in **Figure 4F**, the hepatic mRNAs expression of IFN-γ, TNF-α, IL-10, IL-6, IL-4 and IL-2 were upregulated in the Con A group, while inosine treatment clearly attenuated these changes. However, the expression level of TNF-α and IL-4 did not vary significantly between the Con A group and the Con A+ Inosine group. These results implied that treatment with inosine prevented the inflammatory response.

### Inosine regulated the activation and glucose metabolism of CD4^+^ T cells *in vitro*

**Figure 5:**
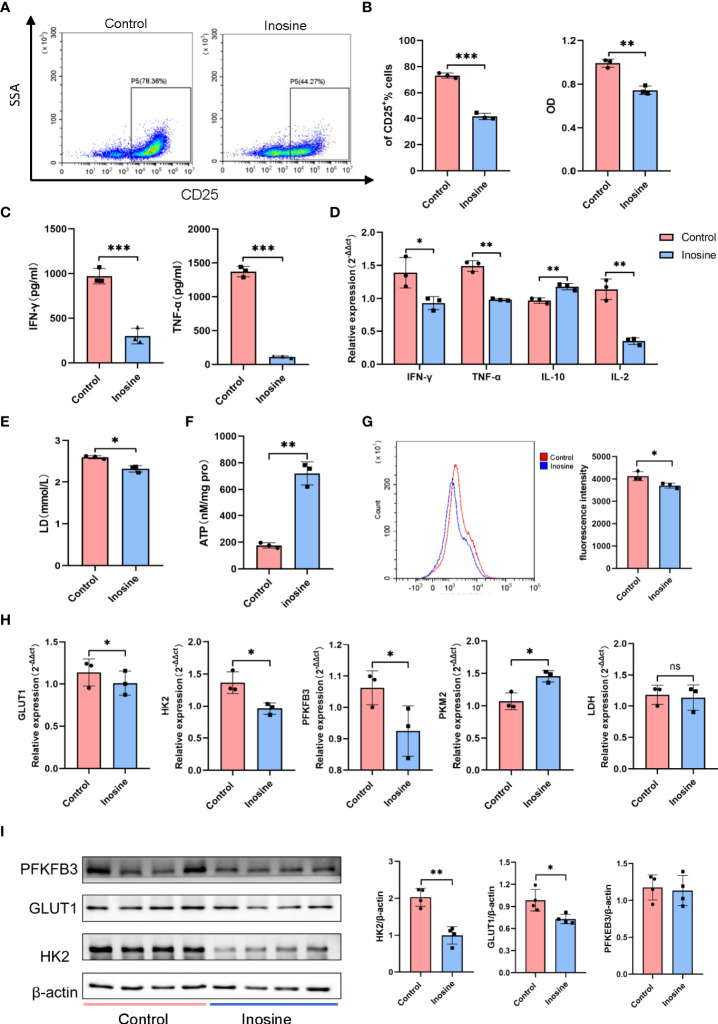
The effect of inosine on CD4^+^ T cells. **(A, B)** Suppression of the activation and proliferation of CD4^+^ T cells by inosine (2 mM): flow cytometric analysis and CCK8 assay. **(C)** Levels of IFN-γ and TNF-α in the culture supernatants. **(D)** The mRNA expression of IFN-γ, TNF-α, IL-10 and IL-2. **(E, F)** Lactic acid level and ATP concentration in the culture supernatant. **(G)** Glucose uptake of CD4^+^ T cells (2-NBDG). **(H)** the mRNA expression of GLUT1, HK2, PK-M2, PFKFB3 and LDH, and relative to β-actin expression. **(I)** Protein expression of PFKFB3, GLUT1 and HK2, and relative to β-actin expression. *p value < 0.05, **p value < 0.01, and ***p value < 0.001.

Con A is a T-cell mitogen that can activate CD4^+^ T cells, followed by an inflammatory reaction. To better explore the effect of inosine on CD4^+^ T cells, CD4^+^ T cells were isolated from mouse spleen by immunomagnetic cell sorting. The purity of CD4^+^ T cells was approximately 99% (**Supplementary Figure S1B**). Then the CD4^+^ T cells were stimulated with plate-bound anti-CD3 mAb and soluble anti-CD28 mAb for 24 h to examine the effects of inosine. The frequency of CD4^+^CD25^+^ cells dramatically increased after TCR simulation (**Supplementary Figure S1C**). Then, we examined the dose dependent effect of inosine on CD25 expression. Treatment with 0.4-2 mM inosine significantly reduced the frequency of CD25^+^ cells and did not exhibit cytotoxicity (**Supplementary Figures S1D, E**). Since CD25 expression was suppressed by approximately 50% by 2 mM inosine without cytotoxicity, we used this concentration for further studies of the suppression of T cell activation (**Figure 5A**). A CCK8 assay was performed to measure the effect of inosine on the proliferation of CD4^+^ T cells. The results showed that inosine treatment significantly suppressed cell proliferation (**Figure 5B**). Next, the IFN-γ and TNF-α levels in the supernatants of cell cultures were determined by ELISA. The results showed that treatment with inosine reduced the release of IFN-γ and TNF-α (**Figure 5C**). To investigate the effect of inosine on cytokine production at the transcriptional level, we detected the mRNA expression levels of IFN-γ, TNF-α, IL-10 and IL-2 by RT-qPCR. As shown in **Figure 5D**, inosine significantly suppressed the IFN-γ, TNF-α and IL-2 expression levels and increased the IL-10 expression level. These data supported that inosine had anti-inflammatory effects on CD4^+^ T cells.

During activation, T cells require various metabolic pathways to carry out their functions. Previous studies have demonstrated enhanced glycolysis in T cells following TLR stimulation. Therefore, we detected the lactic acid level and ATP concentration in the culture supernatants. The results showed that inosine treatment suppressed lactic acid secretion and prompted ATP production (**Figures 5E, F**). Moreover, CD4^+^T cells treated with inosine had significantly lower glucose uptake compared than untreated cells, as indicated by the significant decrease in 2-NBDG (**Figure 5G**). To further investigate glycolysis alterations in CD4^+^ T cells, the expression of glycolytic-related genes and proteins was examined. Compared to the control, inosine treated CD4^+^T cells expressed significantly lower mRNA levels of glucose transporter 1 (GLUT1), hexokinase 2 (HK2) and 6-phosphofructo-2-kinase (PFKFB3) except pyruvate kinase-M2 (PK-M2), all of which are involved in glycolysis (**Figure 5H**). However, lactate dehydrogenase (LDH) mRNA expression levels did not vary significantly between the two groups. Furthermore, the protein expression levels of GLUT1 and HK2 were also significantly downregulated in inosine-treated CD4^+^T cells compared to control cells. The PFKFB3 expression level in the inosine group tended to be similar with that in the control group (**Figure 5I**). In general, these results demonstrated that inosine treatment inhibited glycolysis in CD4^+^ T cells.

## Discussion

Several studies have demonstrated that AIH is related to HLA-DRB1*03:01 and DRB1*04:01 in European populations (3) and DRB1*04:05 in Japanese populations (4–6). In the present study, we showed an association between clinical features of AIH in Chinese patients and HLA-DRB1*04:05, which accounted for 14.9% of the allele frequency in our patients. The specific demographic features of Chinese AIH patients were compared between the DRB1*04:05 positive and DRB1*04:05 negative groups. The serum level of IgG, the positive rate of SLA and the IAIHG score were higher in the HLA-DRB1*04:05 positive group than in the negative group, as previously described (5, 25). Moreover, the time to remission was longer in the HLA-DRB1*04:05 positive group than in the negative group, which may suggest that HLA-DRB1*04:05 was associated with the response to treatment. However, the allele frequency of DRB1*03:01 was 16.9%, which was inconsistent with the previous study (4). This may be due to our limited number of patients. We also compared the clinical characteristics between DRB1*0301 positive and DRB1*0301 negative patients. The results showed that there was almost no association between clinical features of AIH in Chinese patients and HLA-DRB1*03:01 (**Supplementary Table S3**). In addition, there were no healthy controls for HLA haplotyping. Thus, the results could not reveal the predisposition of AIH in Chinese patients with DRB1*03:01, which was also one of the limitations of this study. Although HLA alleles are considered to confer the risk for various autoimmune diseases, the precise mechanism remains unclear. Some studies have demonstrated that autoantigens encoded by HLA genes are expressed on the cell surface and presented to immune cells, which activates the downstream immune process (26). Thus, Chinese AIH patients with DRB1*04:05 have typical clinical traits, probably because of the proteins presented by HLA-DRB1*04:05.

Metabolomics is used to detect small-molecule metabolite profiles to characterize different pathological conditions such as fibrosis and cirrhosis. Most studies have focused on the metabolic fingerprint to find noninvasive biomarkers for the diagnosis and management of autoimmune liver diseases, mainly compared to healthy controls (27). To our knowledge, there have been no previous studies on the associations of serum metabolites and HLA alleles in AIH. Thus, we divided our patients into two groups (DRB1*04:05 positive and negative) and detected serum metabolite profiles. Our results identified 70 metabolites with significant differences between the DRB1*04:05 positive and DRB1*04:05 negative groups. The top 10 most highly altered metabolites between two groups were mainly in nucleotides and their derivates, including inosine, hypoxanthine-9-beta-D-Arabinofuranoside, hypoxanthine and allopurinol. The main pathways involved were the metabolism related such as lipid metabolism, amino acid metabolism, carbohydrate metabolism and nucleotide metabolism. These results revealed the metabolic heterogeneity caused by HLA-DRB1*04:05.

Inosine, as a highly changed metabolite between the two groups, is a natural purine nucleoside composed of hypoxanthine and D-ribose. Previous studies found that inosine is not only a physiological metabolite but also possesses anti-inflammatory and immunoregulatory functions (28, 29). Con A-induced hepatitis is a common experimental acute hepatitis murine model. Consequently, the Con A-induced mouse model was used to detect the effect of inosine treatment. The results demonstrated the protective effect of inosine on Con A-induced liver damage. Inosine treatment obviously reduced the serum ALT and AST levels, which are considered the most informative biochemical markers for diagnosing liver injury. Moreover, treatment with inosine clearly attenuated hepatocyte apoptosis and prevented oxidative stress. H&E staining and TUNEL assays showed that inosine significantly ameliorated the pathological symptoms and reduced the apoptotic cells. Caspase-3, as the final executor of apoptosis, was decreased by inosine treatment, as was NLRP3. MDA, a secondary metabolite produced by free-radical attack, is widely used to reflect the extent of cellular injury (30). Inosine treatment obviously suppressed the elevation of serum MDA levels induced by Con A. Glutathione is a tripeptide that is involved in antioxidant and drug metabolism. The levels of GSH and GSSG are regarded as important indicators of oxidative stress. Treatment with inosine clearly reversed the GSH/GSSG ratio. The expression of oxidative stress genes such as COX-2, iNOS and ACSL4 was suppressed by inosine treatment. Furthermore, the expression of antioxidative stress genes and proteins associated with ferroptosis such as FTH1 and GPX4, was significantly reversed. All these results showed that inosine treatment attenuated hepatocyte apoptosis and increased antioxidant ability in acute severe hepatitis mice.

Con A-induced hepatitis is characterized by the activation of T cells and the release of many cytokines. Our results showed that the frequencies of hepatic CD4^+^CD25^+^ T cells, CD8^+^CD25^+^ T cells, CD4^+^CD69^+^ T cells and CD8^+^CD69^+^ T cells were reduced by inosine treatment. CD25 and CD69 are common activation markers of T cells. In addition, the serum level of and gene expression of proinflammatory cytokines such as IFN-γ, TNF-α, IL-6 and IL-2 were all reduced by the treatment with inosine, while the anti-inflammatory factors such as IL-10 increased. IFN-γ and TNF-α are associated with many inflammatory diseases, including rheumatoid arthritis and systemic lupus erythematosus. IL-6 and IL-2 are related to local or systemic inflammation (31, 32). Thus, the results revealed the inhibitory effect of inosine on immune cells.

CD4^+^ T cells play a crucial role in maintaining immune homeostasis, but their overactivity has been related to the development of many immune-mediated inflammatory diseases, including AIH. Several studies have demonstrated that CD4^+^ T cells are part of the presence in the inflammatory infiltrate in AIH (33). AIH patients often present an impaired T cell number and function (3). Thus, the CD4^+^ T cells were isolated from mouse spleens and stimulated with CD3/CD28. The results showed that inosine treatment suppressed the activation and proliferation of CD4^+^T cells *in vitro*, which is consistent with the *in vivo* results. Additionally, inosine inhibited the release of proinflammatory cytokines, such as IFN-γ, TNF-α and IL-2, from the stimulated CD4^+^ T cells and reduced the related gene expression. Recent advances have shown that T-cell activation and proliferation are supported by metabolic reprogramming (34, 35). Many findings have revealed that aerobic glycolysis is linked to the T cell activation (36, 37). Metabolic inhibitors, especially glycolysis inhibitors, are used to target autoreactive T cells for the treatment of autoimmune diseases (38). Our data newly suggested that inosine could significantly reduce glycolytic genes expression, glucose uptake and the expression of glucose transporters, ultimately leading to a less glycolytic phenotype. This is the first study to reveal alerted glycolysis in CD4^+^ T cells treated with inosine.

This study also has some limitations. The first limitation of our study is that there were no healthy controls for HLA haplotyping, which may not reveal the predisposition of AIH in Chinese patients with HLA-DRB1*04:05. However, the results showed an association between HLA and clinical features of AIH patients. Second, we did not choose the top metabolite to study, because the highly altered metabolites were mainly in nucleotides and their derivates. Inosine, as the top alerted metabolite of nucleotides, has been reported to possess immunomodulatory properties (23). In the future studies, we will explore the role of other metabolites in AIH. In addition, there are some deficiencies in the Con A-induced murine model to completely mimic the pathogenesis of AIH patients. However, Con A-induced liver injury, as a lymphocyte-induced hepatitis murine model, is commonly used to study the pathological mechanisms of autoimmune liver diseases (24).

In conclusion, HLA-DRB1*04:05 is linked to the clinical features of Chinese AIH patients and causes metabolic heterogeneity. Inosine, as a highly altered metabolite, might be involved in the development of AIH through its protective effect on hepatocytes and inhibition of overactivated immune cells, which provide a potential approach for acute form of AIH treatment.

## Data availability statement

The original contributions presented in the study are included in the article/**Supplementary Material**. Further inquiries can be directed to the corresponding authors.

## Ethics statement

The studies involving human participants were reviewed and approved by the Independent Ethics Committee of West China Hospital. The patients/participants provided their written informed consent to participate in this study. The animal study was reviewed and approved by the Animal Ethics Committee of the West China Hospital, Sichuan University.

## Author contributions

LY and XF designed the experiments. FY and LZ performed the experiments and wrote the manuscript. YS and SZ assisted with the experiments. YZ and RM checked the English grammar and polished the English language in the manuscript. All the authors contributed to the article and approved the submitted version.

## Funding

This work was supported by grants from the National Natural Science Foundation of China (No. 82070582) and the 1·3·5 project for disciplines of excellence, West China Hospital, Sichuan University (No. ZYGD20012).

## Acknowledgments

The authors thank Servier Medical Art (http://smart.servier.com) for providing elements of the illustrations and appreciate Core Facilities of West China Hospital for the guidance and help to the experimental technology.

## Conflict of interest

The authors declare that the research was conducted in the absence of any commercial or financial relationships that could be construed as a potential conflict of interest.

## Publisher’s note

All claims expressed in this article are solely those of the authors and do not necessarily represent those of their affiliated organizations, or those of the publisher, the editors and the reviewers. Any product that may be evaluated in this article, or claim that may be made by its manufacturer, is not guaranteed or endorsed by the publisher.
