# Supplementary material for: Metabolic heterogeneity caused by HLA-DRB1*04:05 and protective effect of inosine on autoimmune hepatitis
Source: Front Immunol. 2022 Aug 5;13:982186. doi: 10.3389/fimmu.2022.982186 (PMC9389112; doi:10.3389/fimmu.2022.982186)
Supplement: Supplementary file 1 [file DataSheet_1.docx]

**Supplementary Table 1: Clinical characteristics of AIH patients at diagnosis**

| Features | AIH patients  n=74 |
| --- | --- |
| Age (years) | 53 (25, 75) |
| Gender (M:F) | 12:62 |
| ALT (IU/L) | 209 (22, 1904) |
| AST (IU/L) | 238 (26, 1751) |
| ALP (IU/L) | 148 (59, 598) |
| GGT (IU/L) | 123 (17, 1003) |
| ALB (g/L) | 37.1±5.7 |
| IgG (g/L) | 25.1±8.9 |
| ANA  ＜1:100  ≥1:100 | 15  59 |
| SLA  －  ＋ | 64  7 |
| G  0~2  3~4 | 29  43 |
| S  0~2  3~4 | 42  30 |
| Pretreatment IAIHG score | 14 (10, 24) |

**Supplementary table 2: Clinical characteristics of patients included in the metabolomics**

| Features | AIH patients  n=48 |
| --- | --- |
| Age (years) | 53 (25, 75) |
| Gender (M:F) | 9:39 |
| ALT (IU/L) | 187 (22, 1904) |
| AST (IU/L) | 232 (26, 1585) |
| ALP (IU/L) | 142 (59, 598) |
| GGT (IU/L) | 100 (17, 1003) |
| ALB (g/L) | 37.6±5.8 |
| IgG (g/L) | 24.2 (13.9, 50.2) |
| ANA  ＜1:100  ≥1:100 | 9  39 |
| SLA  －  ＋ | 43  4 |
| G  0~2  3~4 | 21  26 |
| S  0~2  3~4 | 32  15 |
| Pretreatment IAIHG score | 14 (10, 24) |


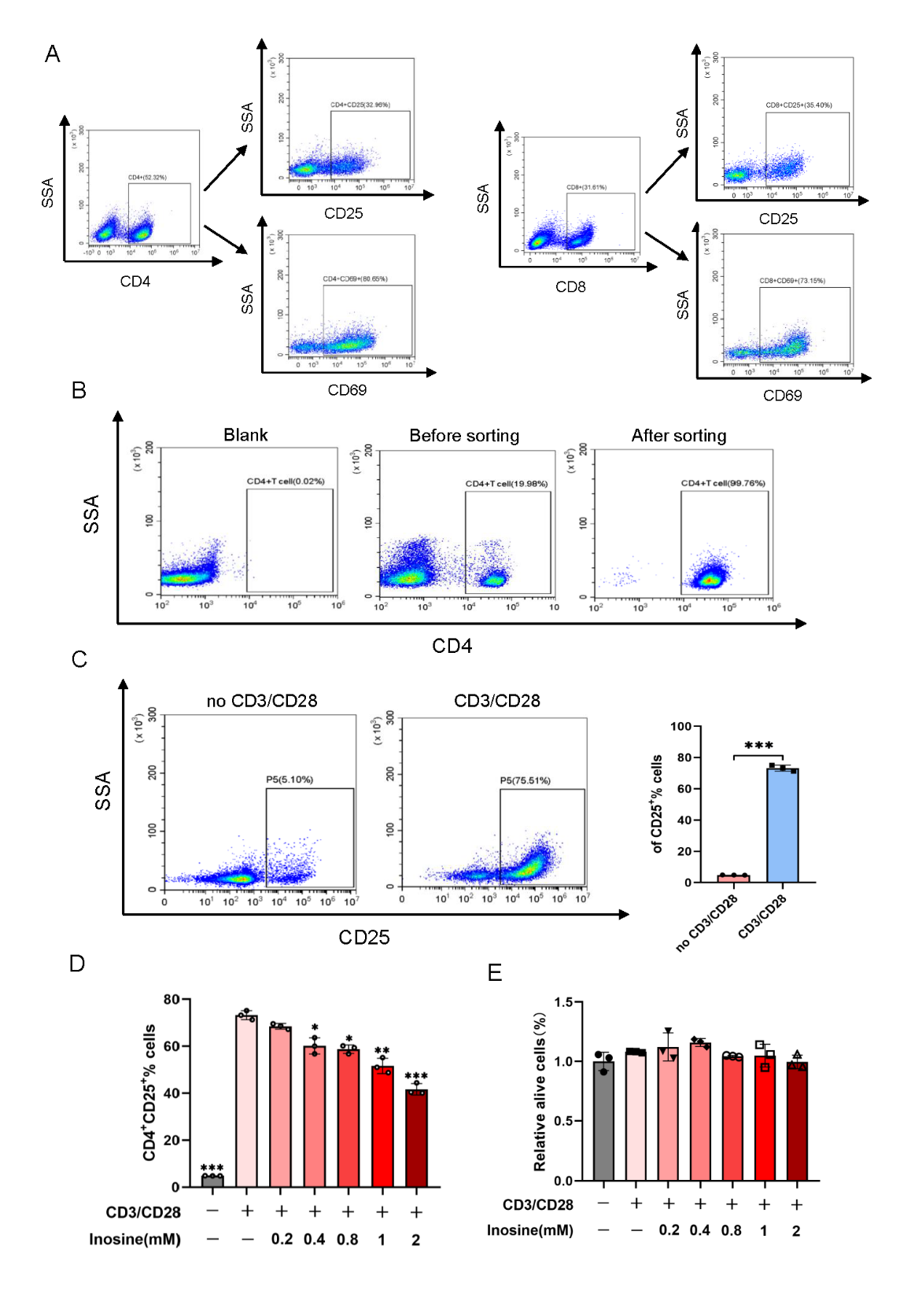


**Supplementary Figure 1: (**A) Gating strategy of CD4^+^CD25^+^ cells, CD4^+^CD69^+^ cells, CD8^+^CD25^+^ cells and CD8^+^CD69^+^ cells. (B) The purity of CD4^+^T cells after sorting. (C) CD25 expression on CD4^+^ T cells after 24 hours of CD3/CD28 stimulation. (D) CD25 expression on CD4^+^ T cells under stimulation with CD3/CD28 and 0.2-2 mM inosine. (E) The relative number of alive cells of CD4^+^ T cells under stimulation with CD3/CD28 and 0.2-2 mM inosine.

**Supplementary Table 3. Clinical characteristics of HLA-DRB1*03:01 positive and HLA-DRB1*03:01 negative AIH patients at diagnosis**

| Features | DRB1*03:01(+)  n=24 | DRB1*03:01(-)  n=50 | P value |
| --- | --- | --- | --- |
| Age (years) | 54±12 | 52±12 | 0.447 |
| Gender (M:F) | 5:19 | 7:43 | 0.682 |
| ALT (IU/L) | 311 (23, 1543) | 183 (22, 1904) | 0.258 |
| AST (IU/L) | 297 (26, 1751) | 220(31, 1585) | 0.640 |
| ALP (IU/L) | 160 (62, 598) | 144 (59, 416) | 0.522 |
| GGT (IU/L) | 132 (17, 1003) | 112 (32, 764) | 0.813 |
| ALB (g/L) | 36.8±6.6 | 37.3±5.3 | 0.717 |
| GLB (g/L) | 38.1±9.2 | 41.3±9.1 | 0.164 |
| IgG (g/L) | 23.4±7.8 | 25.9±9.4 | 0.259 |
| IgM (mg/L) | 1360(411, 3920) | 1650 (251, 8400) | 0.080 |
| ANA  ＜1:100  ≥1:100 | 9  15 | 6  44 | 0.025 |
| SLA  －  ＋ | 24  0 | 40  7 | 0.116 |
| G  0~2  3~4 | 12  11 | 17  32 | 0.159 |
| S  0~2  3~4 | 12  11 | 30  19 | 0.468 |
| Pretreatment IAIHG score | 14 (10, 21) | 14 (10, 24) | 0.374 |
| Complete biochemical remission within 6 months n (%) | 13 (54.2%) | 23 (46.0%) | 1.000 |
| Time to achieve biochemical remission (months) | 4.5 (1.3, 13.3) | 4.9 (0.2, 23.6) | 0.821 |

HLA: human leukocyte antigen, AIH: autoimmune hepatitis, ALT: alanine aminotransferase, AST: aspartate transaminase, ALP: alkaline phosphatase, GGT: gamma-glutamyl transferase, ALB: albumin, GLB: globulin, IgG: immunoglobulin G, IgM: immunoglobulin M, ANA: antinuclear antibody, SLA: soluble liver antigen, G: inflammation grade, S: fibrosis stage, IAIHG: international autoimmune hepatitis group.
